# Correlation between serum 25-hydroxyvitamin D level and diabetic retinopathy: A protocol for systematic review and meta-analysis

**DOI:** 10.1097/MD.0000000000023697

**Published:** 2021-01-29

**Authors:** Lin Chi, Shuang Li, Xinrong Shang, Bo Jiang

**Affiliations:** Beijing Tongren Hospital, Capital Medical University, Beijing 100005, China.

**Keywords:** 25-hydroxy-vitamin D, diabetic retinopathy, meta-analysis, protocol, systematic review

## Abstract

**Background:**

Diabetic retinopathy is a common complication of diabetes with a high incidence, and vitamin D deficiency is associated with diabetic retinopathy. Serum 25-hydroxy vitamin D [25-hydroxy-vitamind, 25 (OH) D], a product of vitamin D in the body, is considered the best indicator of a person's vitamin D nutritional status, and can be determined by measuring the concentration of 25 (OH) D. The purpose of this study is to systematically evaluate the correlation between serum 25-hydroxy vitamin D levels and diabetic retinopathy.

**Methods:**

To search English databases (PubMed, Excerpta Medical Database (Embase), Web of Science, the Cochrane Library) and Chinese databases (Chinese National Knowledge Internet, Development, and Evaluation (CNKI), WanFang, Viper, Chinese Biomedical Literature Database) by computer about Clinical study on the correlation between serum 25-hydroxyvitamin D level and diabetic retinopathy from the establishment of the database to November 2020. Two researchers independently conducted data extraction and literature quality evaluation on the quality of the included studies, and meta-analysis is conducted on the included literatures using Stata12.0 and RevMan5.3 software.

**Conclusion:**

In this study, the correlation between serum 25-hydroxyvitamin D level and diabetic retinopathy was systematically evaluated to provide an evidence-based basis for clinicians.

**Ethics and dissemination:**

Private information from individuals will not be published. This systematic review also does not involve endangering participant rights. Ethical approval was not required. The results may be published in a peer-reviewed journal or disseminated at relevant conferences.

**OSF Registration number:**

DOI 10.17605/OSF.IO/CQY94.

## 1 Introduction

With the change of people's lifestyle and diet, diabetes gradually becomes a global public health problem. The World Health Organization (WHO) reports that the latest global diabetes patients number up to 422 million, which is expected to reach 642 million by 2040, with significant morbidity and mortality worldwide.^[1,2]^ In addition to the harmful effects of diabetes itself, its long-term complications can significantly reduce the quality of lives of people with diabetes. Diabetic patients with uncontrolled or poorly controlled blood glucose have a higher risk of microvascular complications. Diabetic retinopathy (DR) is one of the most serious microvascular complications and a major cause of blindness among working-age people worldwide.^[3,4]^

The combination of vitamin D and the receptor can play a protective role by stabilizing blood glucose, inhibiting renin–angiotensin–aldosterone system, anti-inflammatory, anti-oxidative stress, anti-fibrosis, regulating immunity, and other mechanisms.^[5,6]^ Studies have shown that vitamin D deficiency is correlated with DR to some extent.^[7]^ The serum 25-hydroxyvitamin D [25-hydroxy-vitamind, 25 (OH) D] is a metabolite of vitamin D in the body, which is relatively stable in the body and considered to be the best indicator of the nutritional status of vitamin D. The level of vitamin D in a patient can be determined by measuring the concentration of 25 (OH) D.^[8]^

In recent years, studies on the correlation between serum 25 (OH) D3 concentration and DR have been frequently reported,^[9–11]^ but the results are inconsistent, and the evidence of whether 25 (OH) D3 level is related to diabetic retinopathy is not sufficient. This systematic evaluation aims to evaluate the correlation between serum 25-hydroxyvitamin D level and diabetic retinopathy, and to provide evidence-based evidence for clinicians.

## 2 Methods

### 2.1 Protocol register

This protocol of systematic review and meta-analysis has been drafted under the guidance of the preferred reporting items for systematic reviews and meta-analyses protocols (PRISMA-P).^[12]^ Moreover, it has been registered on open science framework (OSF) (OSF Registration number: DOI 10.17605/OSF.IO/CQY94).

### 2.2 Ethics

Since this is a protocol with no patient recruitment and personal information collection, the approval of the ethics committee is not required.

### 2.3 Inclusion criteria

(1) The literature is case–control study or cross-sectional study.
(2) The research objects are divided into the DR group and the non-diabetic retinopathy (NDR) group.
(3) The objective of each literature study is clear, the statistical method is correct, and the concentration of 25 (OH) D in DR group and NDR group could be provided.
(4) The inclusion of DR group is based on the international clinical classification standard of DR.^[13]^
(5) None of the subjects are artificially added vitamin D as a supplement.
(6) The language limit is Chinese and English.

### 2.4 Exclusion criteria

(1) Repeatedly published papers.
(2) The article whose data cannot be extracted or is incomplete and cannot obtain the data after contacting the author.
(3) Abstracts, comments, abstracts, reviews, case reports, animal experiments, etc.

### 2.5 Retrieval strategy

The computer searches Chinese National Knowledge Internet, Development and Evaluation (CNKI), WanFang, Viper, Chinese Biomedical Literature Database, and Chinese search terms are “*25-Qiang Ji Wei Sheng Su D(25-hydroxyvitamin D)”* or “25 (OH) D (25 (OH) D)*”* and “Tang Niao Bing Shi Wang Mo Bing Bian (Diabetic retinopathy*)*”. Retrieval in English databases including PubMed, Excerpta Medical Database (EMBASE), Web of Science, the Cochrane Library. Search terms in English are *25-hydroxy-vitamin D, 25(OH)D, Vitamin D, Diabetic Retinopathy, DR*. All the Chinese and English literatures on the correlation between serum 25 (OH) D3 concentration and DR are collected from the time of database establishment to November 2020. Take PubMed as an example, and the retrieval strategy is shown in Table 1.

**Table 1:** Search strategy in PubMed database.

Number	Search terms
#1	25-Hydroxy-vitamin D [Title/Abstract]
#2	25(OH)D [Title/Abstract]
#3	Vitamin D [Title/Abstract]
#4	#1 OR #2 OR #3
#5	Diabetic retinopathy [MeSH]
#6	Retinopathy, diabetic [Title/Abstract]
#7	Diabetic retinopathies [Title/Abstract]
#8	DR [Title/Abstract]
#9	#5 OR #6 OR #7 OR #8
#10	#4 AND #9

DR = diabetic retinopathy.

### 2.6 Data filtering and extraction

After the retrieval, 2 researchers independently evaluate the quality of the literature and extract the information according to the inclusion and exclusion criteria. If no agreement could be reached on the judgment results, the third researcher would make a decision after consultation. The extracted information is as follows:

(1) Basic characteristics of the included study, including first author, year of publication, language, study country, study sample size, average age, sex ratio, diabetes type, course of disease, 25 (OH) D concentration, Body Mass Index (BMI), etc.

**Figure 1:**
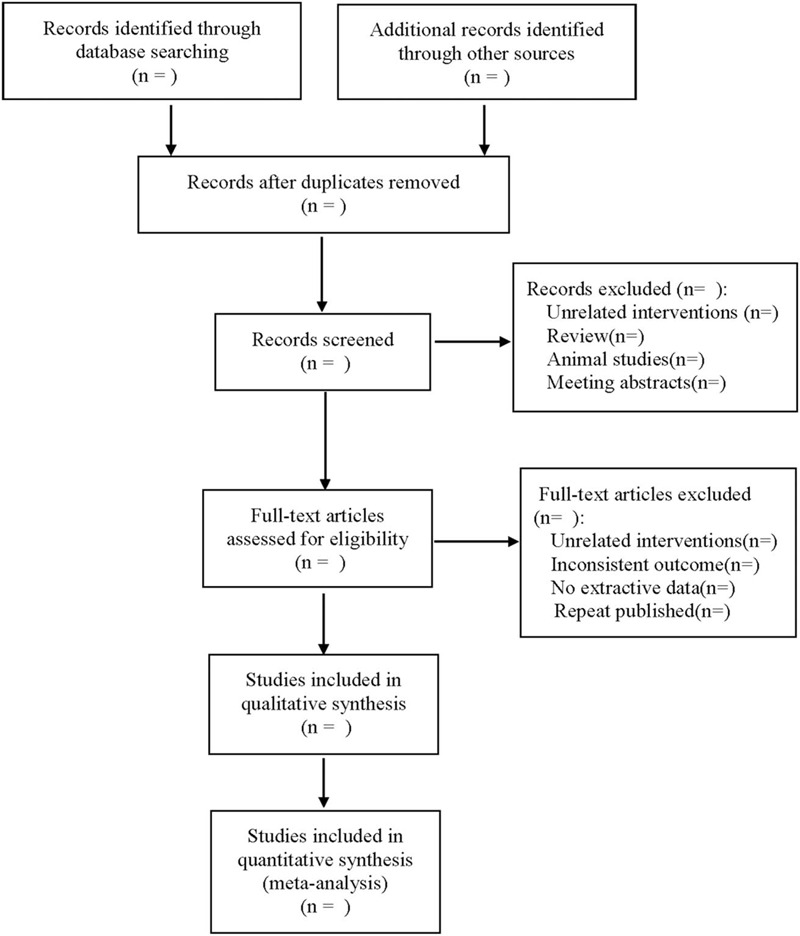
The process of literature filtering.

The key elements of bias risk assessment. The literature selection process is shown in Figure 1.

### 2.7 Literature quality evaluation

The quality of the included research is evaluated^[14]^ according to the Newcastle–Ottawa Scale (NOS), including 3 columns and 8 items, with a total score of 9 points. The score ≥6 points is considered to be relatively high in research quality.

### 2.8 Statistical analysis

#### 2.8.1 Data analysis and processing

RevMan 5.3 software and Stata 12.0 software are used for meta-analysis. Using standardized mean difference (SMD) as the effect indicator, the point estimate and 95%CI of each effect are given, and the heterogeneity among the results is analyzed by chi-square test. At the same time, *I*^2^ index is used to quantitatively judge the heterogeneity. *P* > .10 and *I*^2^ < 50% indicate that little inter-study heterogeneity, then fixed-effect model is adopted for merger. *P* ≤ .10 and *I*^2^ > 50% indicate that the heterogeneity between studies is relatively large, then the source of heterogeneity would be analyzed, and the random effect model would be used for merger. If more than 10 studies are performed, funnel plots will be used to evaluate the existence of publication bias. Additionally, Egger's and Begg's tests will also be used to assess potential publication bias.

#### 2.8.2 Dealing with missing data

If there is missing data in the article, contact the author via email for additional information. If the author cannot be contacted, or the author has lost relevant data, descriptive analysis will be conducted instead of meta-analysis.

#### 2.8.3 Subgroup analysis

In this study, subgroup analysis will be conducted according to different age groups, diabetes type, BMI, and other aspects.

#### 2.8.4 Grading the quality of evidence

We grade the outcome indicators through the Grading of Recommendation Assessment, Development and Evaluation (GRADE).^[15]^ The 2 researchers will evaluate each other and explain the reasons for the downgrade or upgrade results that affect the quality of the evidence, so as to ensure the reliability and transparency of the results. Any differences will be resolved through discussion and consultation with a third investigator until a consensus is reached.

## 3 Discussion

DR is the most common ocular complication of diabetes and one of the important causes of blindness in patients. The early pathological changes include thickening of the basement membrane of capillary endothelial cells, loss of pericytes, and loss of capillary automatic regulation. Then endothelial barrier function is impaired, blood–retina barrier is destroyed, blood components leak out, capillary occlusion, retinal edema. Late angiogenesis, abnormal angiogenesis, and fibrotic hyperplasia are observed.^[16]^ The occurrence of DR is generally believed to be related to the patient's hyperglycemia, which will promote the adhesion of white blood cells to the microvascular epithelial cells, cause cell damage, hemorheology changes, and lead to retinal destruction.^[17]^

Vitamin D is a group of fat-soluble steroid derivatives. In addition to regulating human calcium and phosphorus metabolism, it can also affect the growth and differentiation of a variety of immune cells and play an important role in immune regulation and inflammatory defense.^[18]^ If the level of inflammatory factors in patients is too high, VD can reduce the risk of diabetic microvascular lesions by inhibiting the secretion of inflammatory factors.^[19]^ Studies have shown that insufficient or insufficient serum vitamin D levels can increase insulin resistance and reduce islet cell function.^[20]^ Many studies have shown that in patients with type 2 diabetes mellitus (T2DM), serum 25 (OH) D is negatively correlated with glycosylated hemoglobin (HbA1c) and empty abdominal blood glucose (FPG). The decreased level of 25 (OH) D may be one of the mechanisms for the occurrence of T2DM, and the decreased level of 25 (OH) D may increase the risk of T2DM complicated with peripheral vascular lesions.^[21]^ The reason why vitamin D is not clear to the pathogenesis of DR is probably that serum 25 (OH) D inhibition of vascular endothelial growth factor (VEGF) in the retinal tissue can reduce the apoptosis of vascular endothelial cells caused by the overexpression of VEGF, and the increase of VEGF can enhance the toxicity of blood glucose to neurons, aggravate microvascular complications, accelerate the invasion of vitreous body, and promote the occurrence and development of DR.^[22,23]^

As 25 (OH) D is a metabolite of vitamin D that can be detected in the blood, the detection method is accurate and reliable. For the content is relatively high in the blood, the half-life is long, about 3 weeks, most studies reflect the vitamin D level in the body by measuring its concentration in the serum.^[24]^ This systematic evaluation will evaluate the correlation between serum 25 (OH) D and diabetic retinopathy based on existing evidence, providing an evidence-based basis for the prevention, clinical diagnosis, and treatment of DR monitoring with 25 (OH) D level.

The limitations of this study are as follows: factors such as diet, diabetes treatment, sunlight exposure, and physical activity may have influenced the results. And different 25 (OH) D detection methods may result in clinical heterogeneity. In addition, due to the limitation of language ability, we only search literature in English and Chinese, and may ignore researches or reports in other languages.

## Author contributions

**Data curation:** Lin Chi, Shuang Li, Xinrong Shang.

**Funding support:** Lin Chi.

**Literature retrieval:** Lin Chi and Shuang Li.

**Software operating:** Bo Jiang.

**Software:** Bo Jiang.

**Supervision:** Lin Chi.

**Writing – original draft:** Lin Chi and Shuang Li.

**Writing – review & editing:** Lin Chi and Xinrong Shang.

## Abbreviations

Abbreviations: 25 (OH) D = 25-hydroxy-vitamin D, BMI = Body Mass Index, CNKI = Chinese National Knowledge Internet, Development, and Evaluation, DR = diabetic retinopathy, Embase = Excerpta Medical Database, FPG = empty abdominal blood glucose, GRADE = Grading of Recommendation Assessment, HbA1c = glycosylated hemoglobin, NDR = non-diabetic retinopathy, NOS = Newcastle–Ottawa Scale, OSF = open science framework, PRISMA-P = preferred reporting items for systematic reviews and meta-analyses protocols, SMD = standardized mean difference, T2DM = type 2 diabetes mellitus, VEGF = vascular endothelial growth factor, WHO = World Health Organization.
